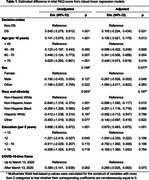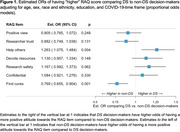# Comparing Research Attitudes in Down Syndrome and Non‐Down Syndrome Research Decision‐makers

**DOI:** 10.1002/alz.090236

**Published:** 2025-01-09

**Authors:** Thuy V Lu, Paola Campos, Sean Leader, Xavier Lee, Helena Xu, Eric Doran, Daniel L Gillen, Joshua D Grill, Ira T. Lott

**Affiliations:** ^1^ University of California, Irvine, Irvine, CA USA; ^2^ Institute for Memory Impairments and Neurological Disorders, University of California, Irvine, Irvine, CA USA; ^3^ California State University, Stanislaus, Turlock, CA USA; ^4^ Cal Poly, San Luis Obispo, San Luis Obispo, CA USA; ^5^ University of Washington, Seattle, WA USA; ^6^ University of California, Los Angeles, Los Angeles, CA USA

## Abstract

**Background:**

Recruitment challenges in people with and without Down syndrome (DS) can delay research progress and risk sample bias. This study identified and quantified differences in research attitudes across populations of research enrollment decision‐makers for individuals with and without DS.

**Method:**

We compared scores on the Research Attitudes Questionnaire (RAQ) of individuals enrolled in two recruitment registries: the UCI Consent to Contact [C2C (N = 4818)] and DS‐Connect (N = 976). We compared total RAQ scores using linear regression. We assessed item‐level RAQ differences using proportional odds regression.

**Result:**

Mean total RAQ scores were not statistically different between DS (DS‐Connect) and non‐DS (C2C) decision‐makers, adjusting for age, sex, race and ethnicity, education, and COVID‐19‐time frame (Est. Diff = 0.11, 95% CI: ‐0.22, 0.43; p = 0.531) (see Table 1). In prespecified item‐level comparisons we did, however, find evidence of differential attitudes on item‐level RAQ scores (see Figure 1). Specifically, DS participants had an increased odds of a more favorable response to the question of responsibility to help others (DS vs non‐DS: OR = 1.26, 95% CI: 1.08, 1.48) and a decreased odds of a more favorable response to the question regarding belief that medical research would find cures for major diseases during their lifetime (DS vs non‐DS: OR = 0.77, 95% CI: 0.66, 0.90).

**Conclusion:**

Our findings provide a framework for researchers to develop precise strategies for recruiting DS and non‐DS individuals into clinical research. The apparent differences in particular research attitudes across these two populations warrant further investigation to instruct these strategies.